# Data for the calculation of an indicator of the comprehensiveness of conservation of useful wild plants

**DOI:** 10.1016/j.dib.2018.11.125

**Published:** 2018-12-04

**Authors:** Colin K. Khoury, Daniel Amariles, Jonatan Stivens Soto, Maria Victoria Diaz, Steven Sotelo, Chrystian C. Sosa, Julian Ramírez-Villegas, Harold A. Achicanoy, Nora P. Castañeda-Álvarez, Blanca León, John H. Wiersema

**Affiliations:** a International Center for Tropical Agriculture (CIAT), Km 17, Recta Cali-Palmira, Apartado Aéreo 6713, 763537 Cali, Colombia; b United States Department of Agriculture, Agricultural Research Service, National Laboratory for Genetic Resources Preservation, 1111 South Mason Street, Fort Collins, CO, 80521, USA; c CGIAR Research Program on Climate Change, Agriculture and Food Security (CCAFS), Km 17, Recta Cali-Palmira, Apartado Aéreo 6713, 763537 Cali, Colombia; d Global Crop Diversity Trust, Platz der Vereinten Nationen 7, 53113 Bonn, Germany; e Museo de Historia Natural Universidad Nacional Mayor de San Marcos, Av. Arenales 1256, Apartado 14-0434, Lima-14, Peru; f Department of Geography and the Environment, The University of Texas at Austin, 305 E. 23rd Street, CLA building, main office- CLA 3.306, Austin, TX 78712, USA; g United States Department of Agriculture, Agricultural Research Service, National Germplasm Research Laboratory, Building 003, BARC-West, Beltsville, MD 20705-2350, USA

**Keywords:** Bioclimatic variables, Biodiversity indicators, Eco-geographic predictors, Economic plants, Occurrence data

## Abstract

The datasets and code presented in this article are related to the research article entitled “Comprehensiveness of conservation of useful wild plants: an operational indicator for biodiversity and sustainable development targets”^1^. The indicator methodology includes five main steps, each requiring and producing data, which are fully described and available here. These data include: species taxonomy, uses, and general geographic information (dataset 1); species occurrence data (dataset 2); global administrative areas data (dataset 3); eco-geographic predictors used in species distribution modeling (dataset 4); a world map raster file (dataset 5); species spatial distribution modeling outputs (dataset 6); ecoregion spatial data used in conservation analyses (dataset 7); protected area spatial data used in conservation analyses (dataset 8); and countries, sub-regions, and regions classifications data (dataset 9). These data are available at http://dx.doi.org/10.17632/2jxj4k32m2.1. In combination with the openly accessible methodology code (https://github.com/CIAT-DAPA/UsefulPlants-Indicator), these data facilitate indicator assessments and serve as a baseline against which future calculations of the indicator can be measured. The data can also contribute to other species distribution modeling, ecological research, and conservation analysis purposes.

**Specifications table**

**Table:** 

Subject area	Biological science, Environmental science
More specific subject area	Conservation indicators, Conservation science, Crop wild relatives, Distribution modeling, Economic botany
Type of data	Data spreadsheets, shapefile, raster files, R code
How data was acquired	Compiled from open access databases and processed.
Data format	Processed (Datasets 1-5, 7-9); Processed and analyzed (Dataset 6)
Experimental factors	None
Experimental features	Compiled and processed taxonomic, uses, and general geographic information spreadsheet (dataset 1), compiled and processed occurrence data spreadsheets (dataset 2), compiled and processed global administrative areas shapefile (dataset 3), compiled and processed eco-geographic predictors raster files (dataset 4), compiled and processed world map raster file (dataset 5), calculated raster files and validation data (dataset 6), processed raster files (datasets 7 and 8), compiled and processed countries and regions classifications spreadsheets (dataset 9).
Data source location	Global
Data accessibility	The data presented in this article is freely and publicly available for any academic, educational, and research purpose. Public repository (https://doi.org/10.17632/2jxj4k32m2.1), code at https://github.com/CIAT-DAPA/UsefulPlants-Indicator
Related research article	Khoury, C.K., Amariles, D., Soto, J.S., Diaz, M.V., Sotelo, S., Sosa, C.C., Ramírez-Villegas, J., Achicanoy, H.A., Velásquez-Tibatá, J., Guarino, L., León, B., Navarro-Racines, C., Castañeda-Álvarez, N.P., Dempewolf, H., Wiersema, J.H., Jarvis, A. 2018. Comprehensiveness of conservation of useful wild plants: an operational indicator for biodiversity and sustainable development targets. Ecological Indicators 98: 420-429. https://doi.org/10.1016/j.ecolind.2018.11.016

**Value of the data**

• The data and code can be used to calculate the comprehensiveness of conservation of wild plant species - *ex situ*, *in situ*, and in combination.
• The data can be used as a baseline against which to measure future indicator results, in order to mark progress toward biodiversity conservation and sustainable development targets, including the Convention on Biological Diversity (CBD), Strategic Plan for Biodiversity 2011-2020, Aichi Biodiversity Target 13 [2] and Global Strategy for Plant Conservation (GSPC) Target 9 [3], United Nations Sustainable Development Goal (SDG) 2.5 [4], and Article 5 of the International Treaty on Plant Genetic Resources for Food and Agriculture (ITPGRFA) [5].
• The data can be used for species distribution modeling and adapted for other biological, ecological, and conservation analyses.

## 1 Data

### 1.1 Dataset 1

Species taxonomy, uses, and general geographic information. This dataset is comprised of one excel file with five sheets. Three of these sheets represent raw data from the USDA-Agricultural Research Service GRIN-Global World Economic Plants (WEP) database [6], for all 15,768 taxa included in the database. The first sheet includes a species identifier, species taxonomic names, and species name authors. The second sheet contains the species identifier, taxonomic name, and primary and secondary use categories assigned to the species. Note that species can be assigned to more than one use (and thus more than one row). The third sheet lists the country distributions of the species. Note that species can occur in more than one country (and thus more than one row). The fourth sheet contains the SQL code used to query WEP for the results contained in sheets 1-3. The final sheet contains the selected list of 6941 taxa used in the “*Comprehensiveness of conservation of useful wild plants*” indicator methodology, including the GBIF standardized taxonomic name. Uses for each species are provided in columns without hierarchy (i.e., all uses have equal importance or value in WEP).

### 1.2 Dataset 2

Species occurrence data. This dataset is one zipfile, within which are included the processed (cleaned) occurrence data for all assessed species for which data was available, with one excel file (.csv) for each species, organized and listed by the species identifier. The data include coordinate fields, country iso3 codes, year collected, occurrence type (G, H), and native status (N for native, I for introduced). These data were compiled from the Global Biodiversity Information Facility [7], the Genesys plant genetic resources portal [8], and the Global Crop Wild Relative Occurrence Database [9], with species names standardized against the GBIF Backbone Taxonomy, using the GBIF Species Lookup Tool [10] and the GBIF Species API v1 [11]. Only coordinates located on land and collected more recently than 1950 (or unknown collection date) are included. The dataset also includes one processed counts.csv file, which shows an example of the structure of the summary file for each species needed to run further steps in the analysis.

### 1.3 Dataset 3

Global administrative areas spatial file. This dataset is an R object file (.RDS) comprised of a shapefile (.shp) containing spatial information for all country level administrative areas worldwide [12].

### 1.4 Dataset 4

Eco-geographic predictors used in species distribution modeling. This dataset is an R object file (.RDS) comprised of all 26 global raster files (.tiff) of the eco-geographic predictors used as inputs for species distribution modeling, at 2.5 arc minutes resolution (approximately 5 km at the equator). These predictors include 19 bioclimatic variables, plus solar radiation, wind speed, and water vapor pressure, derived from WorldClim version 2 [13], and altitude, from the CGIAR-CSI dataset based on the NASA Shuttle Radar Topography Mission (STRM) data [14]. Slope and aspect are also included, calculated from the altitude dataset using the terrain function in R package raster [15].

### 1.5 Dataset 5

World map spatial data. This dataset is one zipfile, within which are two 2.5 arc minute resolution raster files (.tif) outlining all land areas worldwide.

### 1.6 Dataset 6

Species-level spatial distribution modeling outputs. This dataset is one zipfile, within which are included the species distribution model results for all assessed species, with a folder for each species organized by the species identifier code. The final validated MaxEnt presence/absence raster (.tiff) is included for all species whose model passed all validation metrics. The CA50 model is included for all species with occurrence data. A model validation metrics file (.csv) is also included.

### 1.7 Dataset 7

Ecoregion spatial data used in the species conservation analysis. This dataset is a global spatial layer (raster in .tif format) containing 867 distinct terrestrial ecoregions [16], rasterized and resampled at a resolution of 2.5 arc minutes to align with the eco-geographic predictors.

### 1.8 Dataset 8

Protected area spatial data used in the species conservation analysis. This dataset is a global spatial layer (raster in .tif format) rasterized from the World Database of Protected Areas (WDPA) dataset [17], including only those terrestrial and coastal reserves marked as designated, inscribed, or established. The dataset was resampled at a resolution of 2.5 arc minutes to align with the eco-geographic predictors. Raster data is 1=protected area or 0 =not protected area.

### 1.9 Dataset 9

Countries, sub-regions, and regions classifications data. This dataset is comprised of one zipfile, within which are seven spreadsheets (.csv) containing country names, country codes, continents, sub-regions, and regions, at United Nations statistics division standard classification [18], [19]. The data also includes spreadsheets listing the counts of species per country and region, as well as the codes of the species native to each country and region, as derived from the WEP database.

All code implemented in our analysis is available at: https://github.com/CIAT-DAPA/UsefulPlants-Indicator.

## 2 Experimental design, materials, and methods

The datasets presented here are inputs and outputs of a methodology that measures the extent of conservation of useful wild plant species in genebanks and other *ex situ* living plant repositories as well as in protected areas, and then combines the species-level results to create a “*Comprehensiveness of conservation of useful wild plants*” indicator at various scales [1]. The methodology includes five main steps, each with data provided in this article, explained below: 1. species names, uses, and geographic information, 2. species occurrence data, 3. species distribution modeling, 4. species conservation analyses, and 5. indicator calculation.

### 2.1 Species names, uses, and geographic information

We extracted species names (i.e., taxonomy), socioeconomic and cultural use information, and geographic data (i.e., countries where the species is listed as occurring) from the USDA-Agricultural Research Service GRIN-Global World Economic Plants (WEP) database [6]. From the full WEP dataset, we selected those plants with the uses that best aligned with our understanding of the intention of the wording of global biodiversity conservation targets related to useful plants [2], [3], [4], [5]. We excluded from the analysis weeds, poisonous plants, and ornamentals, as well as plants listed only as having potential (rather than confirmed) utility as genetic resources. After further filtering to remove species that are strictly cultivated, we selected a total of 6941 distinct socioeconomically and culturally valuable wild plant species for the analysis, the majority of which have been attributed with more than one use. Species taxonomy, uses, and general geographic data for the entire WEP database as well as for the 6941 selected species are available in *dataset 1*.

### 2.2 Species occurrence data

We gathered data from the Global Biodiversity Information Facility [7], the Genesys plant genetic resources portal [8], and the Global Crop Wild Relative Occurrence Database [9]. For each database, we standardized species names against the GBIF Backbone Taxonomy, using the GBIF Species Lookup Tool [10] and the GBIF Species API v1 [11].

In preparation for the conservation analysis, we classified each occurrence record according to whether it would be used only as an input into the species distribution modeling (labeled H, as most of these records source from herbarium records), or whether it would also be considered a ‘site where collected’ location of an existing living plant conservation repository accession (labeled G, as most records source from genebanks). For GBIF, this classification was performed by filtering the “Basis of Record” field, assigning “living specimen” as G, with the other pertinent categories (observation, literature, preserved specimen, human observation, machine observation, material sample, and unknown) assigned as H. All records in Genesys were assigned G, and records from the Global Crop Wild Relative Database had already been categorized appropriately. These records are available per species in *dataset 2*.

To further refine (i.e., “scrub”) the occurrence data in preparation for species distribution modeling, we removed any coordinates located in bodies of water, as well as those records found outside the native range as defined by WEP (using the global administrative areas spatial file in *dataset 3*). We also included only those reference occurrences collected from 1950 to the present (as well as records without a collecting date), to help ensure that we based distribution modeling on relatively recent data and to align this occurrence information with the time period of the climatic information used in the distribution modeling. These scrubbed records are available per species in *dataset 2*.

### 2.3 Species distribution modeling

We chose the following eco-geographic predictor data inputs based on their use in recent publications, ongoing curation, and global coverage: 19 bioclimatic variables, plus solar radiation, wind speed, and water vapor pressure, derived from WorldClim version 2 [13], and altitude, from the CGIAR-CSI dataset based on the NASA Shuttle Radar Topography Mission (STRM) data [14]. We also included slope and aspect, calculated from the altitude dataset using the terrain function in R package raster [15]. All eco-geographic predictors were prepared at a spatial resolution of 2.5 arc minutes (approximately 5 km at the equator) (Table 1) (*dataset 4*).

**Table 1:** Eco-geographic predictors.

Variable	Description	Unit	Name of dataset	Source
1	Annual Mean Temperature	ºC	wc2.0_bio_2.5m_01.tif	WorldClim v2
2	Mean Diurnal Range (Mean of monthly (max temp - min temp))	ºC	wc2.0_bio_2.5m_02.tif	WorldClim v2
3	Isothermality (BIO2/BIO7) (* 100)	N/A	wc2.0_bio_2.5m_03.tif	WorldClim v2
4	Temperature Seasonality (standard deviation *100)	ºC	wc2.0_bio_2.5m_04.tif	WorldClim v2
5	Max Temperature of Warmest Month	ºC	wc2.0_bio_2.5m_05.tif	WorldClim v2
6	Min Temperature of Coldest Month	ºC	wc2.0_bio_2.5m_06.tif	WorldClim v2
7	Temperature Annual Range (BIO5-BIO6)	ºC	wc2.0_bio_2.5m_07.tif	WorldClim v2
8	Mean Temperature of Wettest Quarter	ºC	wc2.0_bio_2.5m_08.tif	WorldClim v2
9	Mean Temperature of Driest Quarter	ºC	wc2.0_bio_2.5m_09.tif	WorldClim v2
10	Mean Temperature of Warmest Quarter	ºC	wc2.0_bio_2.5m_10.tif	WorldClim v2
11	Mean Temperature of Coldest Quarter	ºC	wc2.0_bio_2.5m_11.tif	WorldClim v2
12	Annual Precipitation	mm	wc2.0_bio_2.5m_12.tif	WorldClim v2
13	Precipitation of Wettest Month	mm	wc2.0_bio_2.5m_13.tif	WorldClim v2
14	Precipitation of Driest Month	mm	wc2.0_bio_2.5m_14.tif	WorldClim v2
15	Precipitation Seasonality (Coefficient of Variation)	%	wc2.0_bio_2.5m_15.tif	WorldClim v2
16	Precipitation of Wettest Quarter	mm	wc2.0_bio_2.5m_16.tif	WorldClim v2
17	Precipitation of Driest Quarter	mm	wc2.0_bio_2.5m_17.tif	WorldClim v2
18	Precipitation of Warmest Quarter	mm	wc2.0_bio_2.5m_18.tif	WorldClim v2
19	Precipitation of Coldest Quarter	mm	wc2.0_bio_2.5m_19.tif	WorldClim v2
20	Solar radiation	(kJ m^-2^ day^-1^)	wc2.0_bio_2.5m_20.tif	WorldClim v2
21	Wind speed	(m/s)	wc2.0_bio_2.5m_21.tif	WorldClim v2
22	Water vapor pressure	(kPa)	wc2.0_bio_2.5m_22.tif	WorldClim v2
23	Altitude (90 m resolution in raw data)	m	wc2.0_bio_2.5m_23.tif	SRTM
24	Slope	º	wc2.0_bio_2.5m_24.tif	Calculated from SRTM
25	Aspect (North-South)	N/A	wc2.0_bio_2.5m_25.tif	Calculated from SRTM
26	Aspect (East-West)	N/A	wc2.0_bio_2.5m_26.tif	Calculated from SRTM

These predictor data were used to model species distributions using the Maximum Entropy (MaxEnt) algorithm [20], [21], run in batch mode through the R statistical package dismo [22]. Pseudo-absences were assigned to cells within the 2.5 arc minute resolution world map raster mask available in *dataset 5*, which was derived from the global administrative areas spatial file (*dataset 3*). Valid MaxEnt models, alternative (CA50) outputs, and validation metrics are included in *dataset 6*.

### 2.4 Species conservation analyses

Two of the measures used to assess adequacy of conservation related to environmental diversity [1]. The layer used for estimating these measures contained 867 distinct terrestrial ecoregions [16] as a proxy for ecological and environmental diversity. This data (*dataset 7*) was rasterized using the Polygon to Raster function in ArcGIS [23], with a 2.5 arc minute raster from WorldClim version 1 [24] used to resample the data so as to align with the resolution of the eco-geographic predictors [25]. During rasterization, the cell center function was used, with the polygon overlapping the center of the raster cell being chosen as the assigned ecoregion type.

To measure the extent of species’ representation within protected areas, we used the World Database of Protected Areas (WDPA) [17], selecting terrestrial and coastal reserves marked as designated, inscribed, or established. This data (*dataset 8*) was rasterized using the Polygon to Raster function in ArcGIS, with a 2.5 arc minute raster from WorldClim version 2 [13] used to resample the data so as to align with the resolution of the eco-geographic predictors. During rasterization, the Maximum area function was used, with the most abundant polygon in the raster cell being chosen as the assigned protected area type.

### 2.5 Indicator calculation

Species level results were combined at country, regional, and global scales by selecting only those species listed as native to those scales as listed in WEP. Regional classifications were aligned to the standard country or area codes for statistical use (M49 code) [18], [19], which are available in *dataset 10*.
